# Hepatoid adenocarcinoma of the stomach with liver metastasis mimicking hepatocellular carcinoma: a case report

**DOI:** 10.4076/1757-1626-2-6317

**Published:** 2009-08-11

**Authors:** Chih-Wen Lin, Chia-Chang Hsu, Hong-Cheng Chang, Yu-Chiun Sun, Po-Lin Sun, Chuan-Yan Hsu, Daw-Shyong Perng

**Affiliations:** 1 Division of Hepatogastroenterology, Department of Internal Medicine, E-Da Hospital/I-Shou University1, E-Da Road, Jiau-shu Tsuen, Yan-chau Shiang, Kaohsiung county 82445Taiwan; 2 Department of Pathology, E-Da Hospital/I-Shou University1, E-Da Road, Jiau-shu Tsuen, Yan-chau Shiang, Kaohsiung county 82445Taiwan; 3 Division of Oncology, Department of Internal Medicine, E-Da Hospital/I-Shou University1, E-Da Road, Jiau-shu Tsuen, Yan-chau Shiang, Kaohsiung county 82445Taiwan; 4 Department of Radiology, E-Da Hospital/I-Shou University1, E-Da Road, Jiau-shu Tsuen, Yan-chau Shiang, Kaohsiung county 82445Taiwan

## Abstract

**Introduction:**

Hepatoid adenocarcinoma is a special type of extrahepatic alpha-fetoprotein-producing adenocarcinoma, which has a morphologic similarity to hepatocellular carcinoma. We report a patient with underlying hepatitis B virus infection and hepatoid adenocarcinoma with liver metastasis mimicking hepatocellular carcinoma.

**Case presentation:**

We present the case of a 56-year-old Chinese female with underlying hepatitis B virus infection, who was found to have multiple hepatic tumors by abdominal ultrasound and an elevated level of serum alpha-fetoprotein. Hepatocellular carcinoma was considered based on the image findings, the elevated level of serum alpha-fetoprotein. and underlying hepatitis B virus infection. Moreover, the subsequent endoscopy revealed gastric tumor. However, the tumor histology of the stomach and liver revealed glandular adenocarcinoma with hepatoid foci. The final diagnosis is hepatoid adenocarcinoma of the stomach with liver metastasis.

**Conclusion:**

Hepatoid adenocarcinoma is an aggressive tumor with liver metastasis being the first clinical manifestation of the neoplasm. Hepatoid adenocarcinoma of the stomach with liver metastasis should be considered in older patients with elevated serum alpha-fetoprotein and multiple hepatic tumors with underlying chronic liver disease. An upper gastrointestinal endoscopy should be performed to exclude the possibility of hepatoid adenocarcinoma originating from the stomach to avoid potential misdiagnosis and inappropriate therapy.

## Introduction

Hepatoid adenocarcinoma (HAC) is a special type of extrahepatic adenocarcinoma, which has a morphologic similarity to hepatocellular carcinoma (HCC) [1]. The first case was described by Bourreille et al. in 1970 [2]. HCC and HAC share numerous clinicopathological features like an elevated level of serum alpha-fetoprotein (AFP), hepatoid morphology and immunoreactivity with AFP and polyclonal carcinoembryonic antigen (CEA) [3]. Thereby, HAC of the stomach with liver metastasis may closely mimic and be indistinguishable form HCC [1]. This makes the differential diagnosis challenging, especially, when the primary tumor is unknown and the first diagnosis has to be established by liver biopsy. Herein, we report a rare case of HAC of the stomach with liver metastasis and underlying hepatitis B virus (HBV) infection which was difficult to differentiate from HCC, particularly in a region with a high prevalence of HCC.

## Case presentation

A 56-year-old Chinese female presented on admission to our gastroenterology ward on May 2007, with a history of abdominal dull pain of 2 weeks duration and a weight loss of 6 kg duration a 2-month period. The abdominal pain became progressively worse and most tender in the right upper quarter (RUQ). She had had HBV infection for 15 years but follow up was irregular. She denied alcohol or smoking history. Result of physical examination showed RUQ tenderness and hepatomegaly. Results of laboratory workup revealed aspartate aminotransferase level of 130 IU/L, alanine aminotransferase level of 50 IU/L, total bilirubin level of 0.6 mg/dl and alkaline phosphatase level of 609 IU/L. The level of serum AFP was elevated (9457 ng/ml) but the levels of CEA and carbohydrate antigen 19-9 (CA19-9) were within the normal range. Serum was positive for hepatitis B surface antigen (HBsAg), but negative for HBeAg. The serum anti-HCV was also negative.

**Figure 1.**
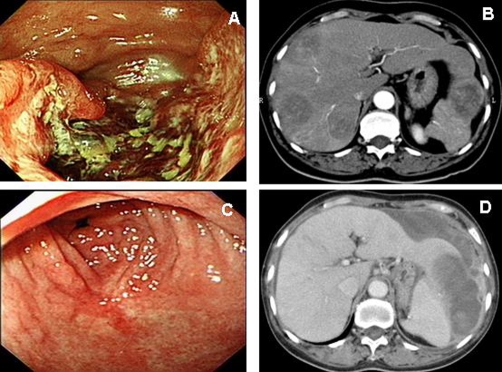
**(A)** Endoscopy reveals a protruding tumor with a central ulceration at the great curvature extending from the low body to antrum of the stomach. **(B)** Abdominal CT shows multiple hepatic tumors in the bilateral lobes of the liver and wall thickening in the stomach. **(C)** Endoscopy reveals complete remission of the gastric tumor after chemotherapy. **(D)** Abdominal CT shows the recurrence of the liver metastases with tumor rupture.

**Figure 2.**
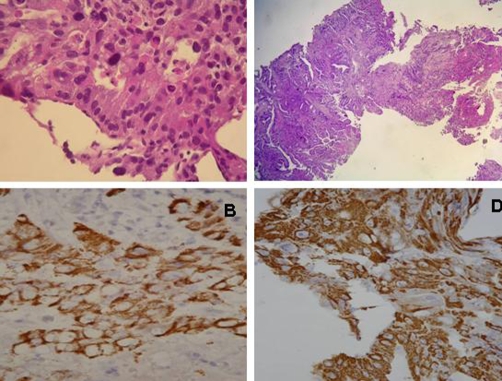
**(A)** Tumor in liver shows glandular structure with eccentrically located nuclei and with hepatoid differentiation. **(B)** Tumor in stomach shows an admixture of tubular adenocarcinoma with hepatoid component. **(C), (D)** Tumors in liver and stomach reactive to hepatocyte paraffin 1 immunohistochemically, suggestive of hepatoid differentiation.

Abdominal ultrasound (US) demonstrated multiple hepatic tumors at the bilateral lobe and mild chronic liver disease. Abdominal computed tomography (CT) showed multiple hepatic tumors sized 3-7 cm in the bilateral lobes of the liver and wall thickening in the stomach (Figure 1A). No cirrhotic change was observed. Based on the image findings, the elevated AFP and underlying HBV infection of the patient, HCC was highly suspected and a US-guided liver biopsy was performed for definite diagnosis (Figure 2A). Subsequent upper GI endoscopy also revealed a 5-cm slight protruding tumor with a central ulceration at the great curvature extending from the low body to antrum of the stomach (Figure 1B).

Gastric biopsy revealed a moderately differentiated tubular adenocarcinoma and poorly differentiated adenocarcinoma morphologically (Figure 2B). However, the histopathological and immunohistochemical (IHC) findings demonstrated that tumor in the stomach is characterized by an admixture of tubular adenocarcinoma with hepatoid component and the tumor in liver showed hepatoid differentiation with identifiable glandular structure (Figure 2A and 2B). The results of IHC study revealed reactivity to Hepatocyte paraffin 1 (Hep Par 1), AFP, and polyclonal and monoclonal CEA in the tumors of both liver and stomach (Figure 2C and 2D). The final diagnosis in this case was HAC of the stomach with liver metastasis. She underwent chemotherapy with Mitomycin-C, 5-fluorouracil and Doxorubicin. The tumor of the stomach was in six courses of complete remission (Figure 1C) and the serum AFP returned to within the normal range after six course chemotherapy. Furthermore, the metastatic liver tumors were partly remission. Careful follow-up of this patient revealed recurrence of the liver metastases with tumor rupture (Figure 1D). The patient died 20 months after disease diagnosis.

## Discussion

AFP is detected in the serum of adults with HCC, hepatoblastoma, those with germ cell tumor (especially yolk sac tumor), and those with non-cancerous liver disease with liver regeneration, such as hepatitis and cirrhosis. AFP-producing malignant tumors have been reported in several different organs such as the lung, pancreas, colon, ovary and urinary bladder [4-8]. The stomach is one of the most common sites affected by these tumors, and the first such case was reported by Bourreille et al. in 1970 [1]. Ishikura et al. proposed the term ‘hepatoid adenocarcinoma of the stomach’ for primary gastric carcinomas characterized by both hepatoid differentiation and the production of large amounts of AFP [9]. The reported incidences of AFP-producing gastric adenocarcinoma have been from 1.3%-15% of all gastric cancers [4,5]. Clinically, the neoplasm is characterized by older age, an aggressive clinical course, and poor survival [1,4-8]. Its biologic behavior is due to its extensive hematogenous metastasis to the liver and to its early involvement of lymph nodes [1,5].

The diagnosis of HAC depends on recognition of the characteristic histological features [5]. The histological finding reveals glandular adenocarcinoma with hepatoid foci [6]. The IHC staining affords more special support but it dose not constitute a definite diagnosis for HAC. The IHC staining has not also been helpful in distinguishing between metastatic HAC and HCC [3]. Demonstration of the production of albumin mRNA provides specific support for a diagnosis of hepatocyte differentiation [10]. It is important to ensure the accurate diagnosis of an AFP-producing HAC of stomach as a result of the extremely poor prognosis.

The resemblance of HAC to HCC may be problematic in the diagnosis of hepatic tumors. In our study, multiple hepatic tumors were the first tumor manifestation and the patient had underlying HBV infection and markedly elevated AFP. Thus, the diagnosis of HCA presenting as hepatic tumors may be a great challenge for the pathologist and clinician, and the differential diagnosis of HAC from HCC is difficult. HCCs arising in non-fibrotic liver and without underlying known risk factors are rare, but according to a European study they may represent up to 13% of HCCs [11]. However, this group often appears as a single tumor [12]. Although multiple hepatic tumors without cirrhotic change were found in our study, HCC had been diagnosed in our case because she had multiple hepatic tumors, underlying HBV infection and markedly elevated AFP. Even if the first diagnosis is established by liver biopsy, misdiagnosis as HCC can still occur because the hepatic tumor bears a striking morphologic similarity to HCC in histology and IHC staining especially when a case happens in a region of high prevalence of HCC such as in Taiwan. Therefore, if the extrahepatic origin is not detected by endoscopy, the misdiagnosis and inappropriate therapy may follow.

In our study, the tumor of both stomach and liver was positive for Hep Par1. Hep Par 1 is a developed monoclonal antibody that reacts with a hepatocyte-specific epitope [13]. An 82% sensitivity and 90% specificity for Hep Par1 have been reported in HCC, suggesting that this antibody might be the most sensitive and specific IHC marker for hepatocyte differentiation [1]. Furthermore, some studies have reported a focal positivity for Hep Par1 in 13% to 85% of carcinoma with hepatoid differentiation [1,14,15]. The presence of Hep Par 1 reactivity in extrahepatic HACs underscores the fact that Hep Par 1 expression is not unique to primary HCCs. Adenocarcinoma with hepatoid features must be considered in the differential diagnosis of Hep Par 1-positive lesions.

## Conclusion

HAC is an aggressive tumor with liver metastasis being the first clinical manifestation of the neoplasm. HAC of the stomach with liver metastasis should be considered in older patients with elevated serum AFP and multiple hepatic tumors with underlying chronic liver disease. An upper GI endoscopy should be performed to exclude the possibility of HAC originating from the stomach. Although rare, this entity deserves wide recognition among pathologists and clinicians to avoid potential misdiagnosis and inappropriate therapy.

## Abbreviations

AFP: Alpha-fetoprotein
CEA: Carcinoembryonic antigen
CT: Computed tomography
GI: Gastrointestinal
HAC: Hepatoid adenocarcinoma
HBV: Hepatitis B virus
HCC: Hepatocellular carcinoma
Hep Par 1: Hepatocyte paraffin 1
IHC: Immunohistochemical
RUQ: Right upper quarter
US: Ultrasound
